# Thrombolysis Combined Therapy Using CuS@SiO_2_-PEG/uPA Nanoparticles

**DOI:** 10.3389/fchem.2021.643411

**Published:** 2021-03-11

**Authors:** Dapeng Fu, Qingbo Fang, Fukang Yuan, Junle Liu, Heyi Ding, Xuan Chen, Chaoyi Cui, Jinhui Ding

**Affiliations:** ^1^ Department of Vascular Surgery, The Second People's Hospital of Anhui, Province, Hefei, China; ^2^ Department of Vascular Surgery, The People’s Hospital of Xinjiang Uygur Autonomous Region, Urumqi, China; ^3^ Department of Vascular Surgery, Fengcheng Hospital of Fengxian District, Shanghai, China; ^4^ Department of Vascular Surgery, Fengcheng Branch, Shanghai Ninth People’s Hospital Affiliated to Shanghai Jiao Tong University School of Medicine, Shanghai, China; ^5^ Department of General Surgery of Xuzhou Central Hospital, Xuzhou, China; ^6^ Department of Vascular Surgery, Shanghai Ninth People’s Hospital, Shanghai Jiao Tong University School of Medicine, Shanghai, China; ^7^ Department of Vascular Surgery, Karamay Central Hospital, Karamay, China

**Keywords:** drug delivery, photothermal therapy, uPA, thrombolysis, silica nanomaterials

## Abstract

Massive hemorrhage caused by the uncontrolled release of thrombolysis drugs is a key issue of thrombolysis therapy in clinical practice. In this study, we report a near-infrared (NIR) light-triggered drug delivery system, i.e., CuS@mSiO_2_-PEG (CSP) nanoparticles, for the loading of a thrombolytic drug (urokinase plasminogen activators, uPA). CSP nanoparticles with the CuS nanoparticles as photothermal agents and mesoporous SiO_2_ for the loading of uPA were synthesized using a facile hydrothermal method. The CSP core-shell nanoparticles were demonstrated to possess excellent photothermal performance, exhibiting a photothermal conversion efficiency of up to 52.8%. Due to the mesoporous SiO_2_ coating, the CSP core-shell nanoparticles exhibited appropriate pore size, high pore volume, and large surface area; thus, they showed great potential to be used as drug carriers. Importantly, the release of uPA from CuS@mSiO_2_-PEG/uPA (CSPA) carriers can be promoted by the NIR laser irradiation. The drug loading content of uPA for the as-prepared NIR-triggered drug delivery system was calculated to be 8.2%, and the loading efficiency can be determined to be as high as 89.6%. Due to the excellent photothermal effect of CSP nanocarriers, the NIR-triggered drug delivery system can be used for infrared thermal imaging *in vivo*. The *in vivo* thrombolysis assessment demonstrated that the NIR-triggered drug delivery system showed excellent thrombolytic ability under the irradiation of an 808 nm laser, showing the combined therapy for thrombolysis. As far as we know, the CSPA core-shell nanoparticles used as NIR-triggered drug delivery systems for thrombolysis have not been reported.

## Introduction

Thrombosis is a disease that often occurs in middle-aged and elderly people. Thrombus is hidden in the human body like a bomb and may explode anytime and anywhere (Di Nisio et al., 2016). If the thrombus travels in the human lungs, pulmonary thrombosis may appear. If it is in the heart, it may cause coronary heart disease and myocardial infarction. In general, once a blood clot occurs, it will pose a great threat to human health (Di Angelantonio et al., 2019). Anticoagulant thrombolytic drugs can be used to treat thrombosis in clinical practice. Since most of these drugs are small molecule drugs, their circulation half-life in the body is very short, which leads to the need for very high doses, leading to lethal side-effects (Engelmann and Massberg, 2013). Urokinase plasminogen activators (uPA) are commonly used thrombolytic drugs clinically. However, the circulation half-life of uPA is very short, thus leading to a poor thrombolysis effect when in a low dose (Jin et al., 2013). Therefore, it is necessary to develop an efficient and safe drug carrier to deliver uPA to treat thrombosis.

With rapidly development of nanotechnology in the past two decades, drug carriers have provided a new idea and strategy for disease diagnosis and treatment (Meng et al., 2016b; Yu et al., 2018; Li et al., 2019). Drug carriers can accumulate in lesion sites to release drugs, reduce side-effects, and improve bioavailability because of their small size, so they were intensively investigated in recent years (Lee et al., 2012). Especially, stimuli-responsive nanocarriers have been demonstrated to be promising drug delivery systems (Wang et al., 2013). There have been various strategies, including light (Liu et al., 2016), temperature (Meng et al., 2016a), biomolecular reactions (Song et al., 2017), and pH (Cohen et al., 2011; Fang et al., 2012) to be reported for the drug delivery for nanocarriers. In these strategies, near-infrared (NIR) light-triggered nanocarriers are a very promising and effective method for drug release due to their high spatial resolutions and minimal damage to the normal tissue (Fang et al., 2012). Importantly, NIR light-triggered nanocarriers usually can be used as both phototherapy agents and drug release system, thus exhibiting a synergistic effect of drug therapy and photothermal therapy for disease therapy (Liu et al., 2015; Liu et al., 2020).

Previous studies have proved that photothermal therapy can effectively dissolve blood clots (Fu et al., 2019; Fang et al., 2020). Photothermal therapy is a technology that utilizes photothermal agents to convert NIR light into heat energy (Tian et al., 2011b; Li et al., 2015; Wang et al., 2019). Due to the facile synthesis, low cost, good photostability, high photothermal effect, and variable chemical formula of copper-based chalcogenide compounds, they have been demonstrated to be a potential class of effective photothermal therapy (PTT) agent (Chen et al., 2013; Zhang et al., 2019). At present, there are several copper-based chalcogenide compounds, such as Cu_9_S_5_ (Tian et al., 2011a), Cu_2-x_Se (Hessel et al., 2011), and CuCo_2_S_4_ (Li et al., 2017), to be developed as PTT agents. The reported copper-based chalcogenides as PTT agents exhibit excellent photothermal performance and photostability, which are the essential properties of PTT agents. The photothermal performance of copper-based chalcogenide nanostructures resulted from the NIR absorption characteristics derived from the free 3-day electronic transition of copper (Ghosh et al., 2016; Li et al., 2017; Peng et al., 2020). Therefore, there is Cu^+^ and Cu^2+^ coexistence in these copper chalcogenides and even a lot of copper vacancy (Li et al., 2015). Although considerable progress has been made in the synthesis of varied kinds of copper chalcogenides, the biocompatibility of these PPT agents is a key issue for clinical applications (Zhou et al., 2016; Coughlan et al., 2017). Mesoporous silica (mSiO_2_) nanoparticles are a kind of drug delivery systems that are widely used in drug delivery due to the excellent biocompatibility, excellent stability, and large specific surface area (Wang et al., 2013; Liu et al., 2015). An effective strategy is coating the nanostructures with mesoporous mSiO_2_ nanoparticles to improve the biocompatibility of copper-based chalcogenide nanostructures (Zhang et al., 2020a; Zhang et al., 2020b). Besides addressing the safety, the mSiO_2_ coating could combine PTT and chemotherapy. Several studies have reported the core-shell nanocomposites with copper-based chalcogenides as the core being photothermal agents and mSiO_2_ nanoparticles and the shell the drug delivery carriers, which combine PTT and drug therapy in a single nanoplatform, exhibiting a synergistic therapeutic effect. For example, Liu et al. reported Cu_2−x_Se@mSiO_2_-PEG nanoparticles with Cu_2−x_Se as photothermal agents and mSiO_2_ as anticancer drug doxorubicin hydrochloride (DOX) carrier for cancer treatment, which showed a synergistic effect as photothermal enhanced chemotherapy (Liu et al., 2015). However, most of the reported core-shell nanostructures are used in cancer therapy. There are few reports on the application of these materials in thrombolysis.

In this study, a NIR light-triggered drug delivery system, i.e., CuS@mSiO_2_-PEG (CSP) nanoparticles, was prepared by a simple method for thrombolysis. CSP nanoparticles with the CuS nanoparticles as the core to be used as photothermal agents and the mesoporous SiO_2_ as the shell for the loading of uPA were synthesized using a facile hydrothermal method. The CSP core-shell nanoparticles were demonstrated to possess excellent photothermal performance, exhibiting a photothermal conversion efficiency of up to 52.8%. Due to the mesoporous SiO_2_ coating, the CSP core-shell nanoparticles exhibited appropriate pore size, high pore volume, and large surface area; thus, they were promising to be used as drug carriers. The drug loading content of uPA for the as-prepared NIR-triggered drug delivery system was calculated to be 8.2%, and the loading efficiency can be determined to be as high as 89.6%. Importantly, the release of uPA from CuS@mSiO_2_-PEG/uPA (CSPA) carriers can be promoted by the NIR laser irradiation. Due to the excellent photothermal effect of CSP nanocarriers, the NIR-triggered drug delivery system can be used for infrared thermal imaging *in vivo*. The *in vivo* thrombolysis assessment demonstrated that the NIR-triggered drug delivery system showed excellent thrombolytic ability under the irradiation of an 808 nm laser. As far as we know, the CSPA core-shell nanoparticles used as NIR-triggered drug delivery systems for thrombolysis have not been reported.

## Experimental Section

### Synthesis of CuS Nanoparticles

All reagents were purchased from China National Pharmaceutical Group and can be directly used. Under vigorous magnetic stirring, sodium citrate (0.1 mmol) and CuCl_2_·2H_2_O (0.1 mmol) were sufficiently dissolved in 50 ml deionized (DI) water. Then, 10 ml sodium sulfide solution ethylenediamine (1 mM) was slowly added to the above solution. The resulting solution was then heated to 80 °C for 5 min and kept for 30 min. The black-green products were collected by centrifugation and washed several times with ethanol and deionized water.

### Synthesis of CSP Core-Shell Nanoparticles

The core-shell nanostructures were synthesized by a modified method (Liu et al., 2015). CTAB (0.5 g) was added to the CuS nanoparticles (20 ml), and then the solution was heated to 40 °C under magnetic stirring for 2 h. NaOH solution (20 mg ml^−1^, 100 μL) and ethanol (3 ml) were added. Then, TEOS (100 μL) was added dropwise. With continuous stirring, the mixed solution was kept at 40 °C for 2 h. After that, PEG-silane (100 μL) was added and kept for another 6 h under stirring. After washing, the CTAB can be removed using ammonium nitrate. Typically, the washed products were fully dispersed in ammonium nitrate (dispersed in 50 ml ethanol, 10 mg ml^−1^), and the mixture solution was kept at 50 °C for 2 h under continuous stirring to remove the CTAB by ion exchange. CSP core-shell nanoparticles were obtained by centrifugation and washing with ethanol and water.

### Characterization

The size, microstructure, and morphology of the as-prepared core-shell nanoparticles were measured by a transmission electron microscope (TEM). X-ray diffraction (XRD) measurement was performed on an X-ray diffractometer (D/max-2550 PC). The UV-vis absorption spectrum with a wavelength scanning range of 400–1,100 nm at room temperature was measured by a UV-vis-NIR spectrophotometer (Shimadzu UV-3600). The content of copper released from the as-synthesized core-shell nanoparticles was determined by ICP-AES (Prodigy, USA). Fourier transform infrared (FTIR) spectrometer (Nicolet 6,700) was used to measure FTIR spectra of the as-synthesized core-shell nanoparticles in KBr pellets. The 808 nm semiconductor laser was used as the light source, and its power can be adjusted externally (0–2 W). In order to measure the photothermal transaction effect, an 808 nm semiconductor laser device was used to irradiate as-synthesized core-shell nanoparticles with varying concentrations at a power density of 0.5 W cm^−2^ for 5 min. The temperature was monitored and recorded by a NIR thermal imaging camera and the images are simultaneously imaged.

### Animal Model

C57 mice were anesthetized. The carotid artery of the mice was separated and was ligated with a surgical line to build carotid artery thrombosis.

### *In Vivo* Thermal Imaging

All the animal procedures were approved by the Guidelines for Care and Use of Laboratory Animals of Karamay Central Hospital. CSP core-shell nanostructures and PBS were intravenously injected into the mice with carotid artery thrombosis. Twenty-four hours after the injection, the mice injected with CSP core-shell nanostructures or PBS were simultaneously irradiated upon the 808 nm laser (0.5 W cm^−2^, 180 s). During the laser irradiation, an infrared camera was used to record the infrared thermal images of the mice in the two groups.

### Thrombolysis *In Vivo*

The mice with the carotid artery thrombosis model were randomly divided into four groups, with six mice in each group. Subsequently, the mice were subjected to different treatments. These four groups are as follows: 1) mice in the control group only irradiated with the 808 nm laser with a power density of 0.5 W cm^−2^ for 5 min (Control); 2) mice injected with CSP core-shell nanostructures dispersed in saline and then irradiated with the same 808 nm laser (CSP + NIR); 3) mice injected with CSPA core-shell nanostructures dispersed in saline without NIR irradiation (CSPA); 4) mice injected with CSPA core-shell nanostructures dispersed in saline with the same 808 nm laser (CSPA + NIR). Twenty-four hours after the above-indicated treatments, carotid artery thrombosis was taken out for histological analysis.

### Histological Analysis

After the indicated treatments, blood vessels at the thrombus and major organs were harvested. The histological examination was then carried out through microscopic imaging.

## Results and Discussion

The NIR-triggered drug delivery system is made of CSP core-shell nanoparticles. These CSP nanoparticles were prepared by a three-step hydrothermal synthesis method. CTAB was used as a soft template in the procuring of mSiO_2_ coating on the surface of CuS. mSiO_2_ coating grew around the CTAB template due to electrostatic interactions. Supplementary Figure S1 shows the size and zeta potential of nanoparticles during the synthesis of CSP core-shell nanoparticles. CuS nanoparticles were prepared in the presence of citric acid. The citric acid ligand makes the CuS particles become negatively charged. As the hydrolysate of TEOS shows a negative charge, the CuS nanoparticles cannot be directly coated by the mSiO_2_ shell. CTAB was coated around the CuS nanoparticles, making the CuS nanoparticles become positively charged. Then, the PEG was used to modify the biocompatibility of the CSP nanoparticles as CTAB has been demonstrated to be toxic.

**FIGURE 1:**
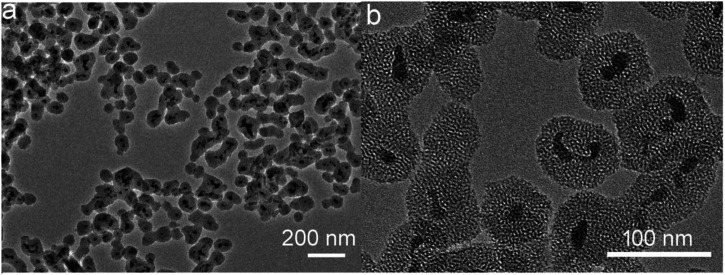
Low-resolution **(A)** and high-resolution **(B)** TEM images of CSP core-shell nanoparticles.

The morphology and size of the synthesized CSP core-shell nanoparticles were measured by TEM. Figure 1A shows the low-resolution TEM image of CSP core-shell nanoparticles. The TEM image showed that CuS nanoparticles were completely coated by SiO_2_ shell. The thickness of the SiO_2_ shell was about 11 nm, and the mean size of CSP core-shell nanoparticles was about 39 nm (Supplementary Figure S1), which was very suitable for PTT agents as biomaterials with such size usually possess long blood circulation time. Figure 1B exhibits the high-resolution TEM image of CSP core-shell nanoparticles. It can be clearly seen that the SiO_2_ shell was mesoporous. Powder XRD was then used to evaluate the crystal structure of the CSP nanoparticles. As shown in Supplementary Figure S2, the main peaks of the XRD patterns of the sample can be matched with the standard CuS hexagonal phase (JCPDS no. 79-2321), demonstrating that we have successfully prepared the CSP nanoparticles with high purity. In addition, there was a wide peak in the XRD patterns of the products, indicating the coating of amorphous silica. These results confirmed the successful formation of CSP nanostructures. To improve the biocompatibility of CuS@mSiO_2_ core-shell nanoparticles, PEG was used to be grafted with mSiO_2_ shell. Therefore, the CSP nanoparticles do not need to undergo any further surface modification. Moreover, the CSP nanoparticles showed excellent dispersion with no change when dispersed in water for 2 weeks and can remain unchanged when dispersed in PBS solution for 7 days. Supplementary Figure S3 shows the FTIR spectrum of CuS@mSiO_2_ core-shell nanoparticles after PEG grafting. The peak at 2925 cm^−1^ for the stretching vibration of methylene (CH_2_) of PEG and the peak at 1,349 cm^−1^ for the deformation vibration of PEG’s backbone indicated that PEG was successfully grafted. According to the above results, CSP core-shell nanoparticles were successfully prepared.

**FIGURE 2:**
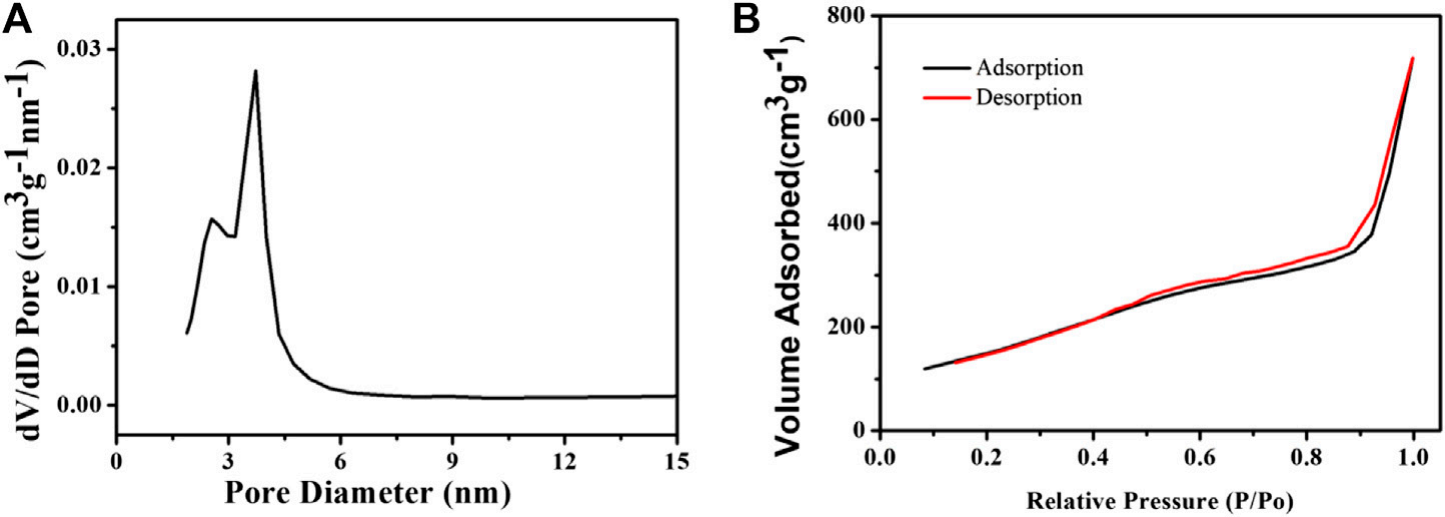
**(A)** Pore size distribution and **(B)** N_2_ adsorption/desorption isotherms of the as-prepared CSP core-shell nanostructures.

The drug carrier requires a suitable pore size and a large specific surface area. We then measured the pore size distribution and the Brunauer–Emmett–Teller (BET) surface area. After removing the CTAB, the pore size of CSP core-shell nanoparticles was measured to be 3.4 nm, which is fit for the loading of thrombolytic drugs, i.e., uPA. As we can see from Figure 2A, the pore size distribution of the as-prepared CSP core-shell nanostructures exhibited a sharp peak at a mean value of 3.2 nm, and the BJH pore volume for CSP core-shell nanostructures was calculated to be 0.713 m^3^ g^−1^. In addition, the BET surface area for CSP core-shell nanostructures was measured to be 712 m^2^ g^−1^ (Figure 2B). Thus, CSP core-shell nanoparticles possessed appropriate pore size, high pore volume, and a large surface area, showing great potential for drug loading.

**FIGURE 3:**
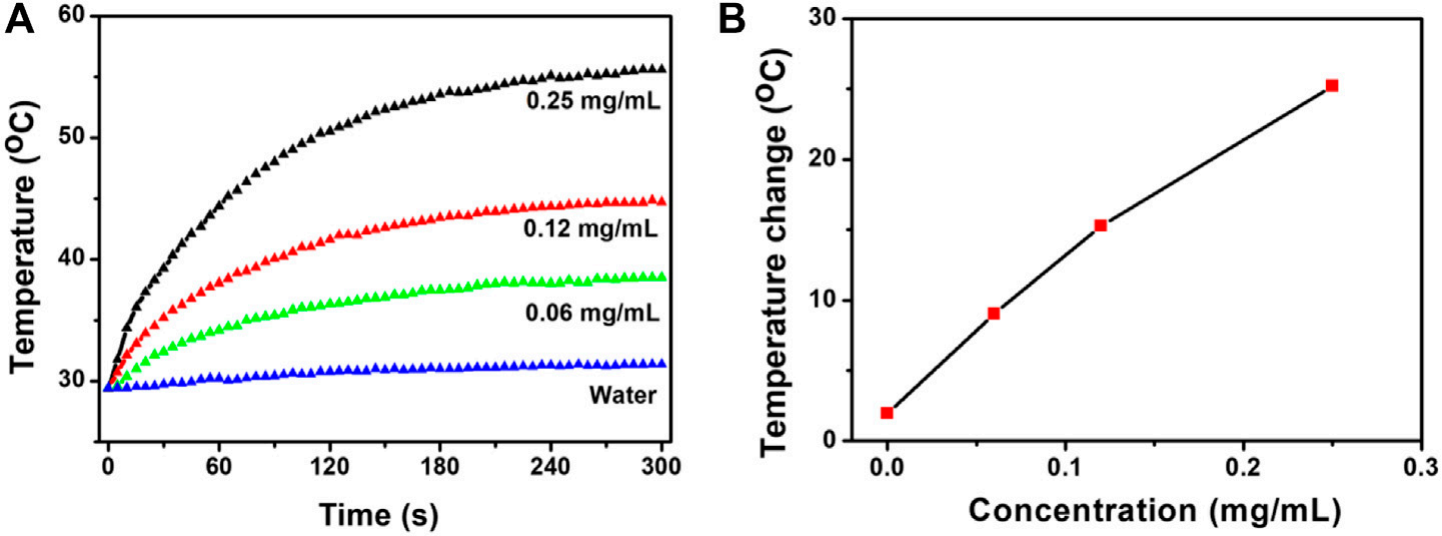
**(A)** Photothermal effect of CSP core-shell nanostructures with varying concentrations under the irradiation of 808 nm lasers. **(B)** Concentration-temperature curves from panel **(A)**.

Based on the characteristics of the NIR absorption properties of CSP core-shell nanostructures (Supplementary Figure S4), we chose 808 nm lasers as the light source to study the photothermal effect of the CSP nanostructures. The extinction coefficient of the CSP nanoparticles at 808 nm was measured to be 14.1 L g^−1^ cm^−1^ by determining the core-shell nanoparticle concentration via ICP-AES. The value was equal to that of copper-based chalcogenide compounds (Zhang et al., 2020a). We first evaluated the photothermal effect of the CSP core-shell nanostructures with varying concentrations (0–0.25 mg/ml) under the irradiation of an 808 nm laser (0.5 W cm^−2^). Obviously, the CSP core-shell nanoparticles showed a concentration-dependent photothermal effect (Figures 3A,B). With no CSP nanoparticles added, the water temperature increased by less than 1 °C under the irradiation of the 808 nm laser. The temperature of a 0.06 mg ml^−1^ solution of CSP nanoparticles was increased by 8.6 °C under the same power density (0.5 W cm^−2^) of the 915 nm laser. With the concentration increased to 0.25 mg ml^−1^, the temperature of CSP core-shell nanostructures was increased by 27.3 °C, which is enough for photothermal triggered drug release. Therefore, CSP core-shell nanostructures showed excellent photothermal effect upon the irradiation of an 808 nm laser.

Photothermal conversion efficiency is one of the key indicators for evaluating photothermal agents. In order to better evaluate the photothermal performance of CSP core-shell nanostructures, the photothermal conversion efficiency of CSP core-shell nanostructures was then measured and calculated by an improved method similar to CuCo_2_S_4_ nanocrystals (Li et al., 2017). The photothermal conversion efficiency, η_T_, was calculated by Eq. 1:

$$\eta_{T}=\frac{\text{hA}\left({\text{T}}_{\max}-{\text{ T}}_{\text{amb}}\right)-Q_{0}}{I\left(1-10^{-A_{\lambda }}\right)}.$$ (1)

**FIGURE 4:**
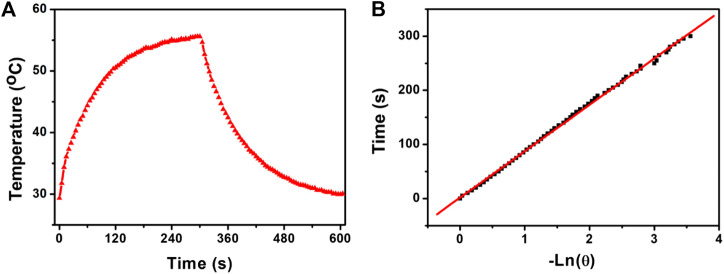
**(A)** Photothermal effect of CSP core-shell nanostructures with a concentration of 0.12 mg/ml under the irradiation of the 808 nm laser. **(B)** Time constant of the system.

In Eq. 1, *I* is the laser power (in mW, 180 mW). A_λ_ is the absorbance (0.54539) of the photothermal agents at the excitation wavelength of the laser. h is the heat transfer coefficient. A is the surface area of the container for the photothermal agents. T_max_ is the maximum temperature of the photothermal agents under the irradiation of the laser. T_amb_ is the ambient temperature. (T_max_ − T_amb_) was 26.3 °C from Figure 4A. Q_0_ is the rate of heat input due to light absorption in the solution (in mW). hA can be obtained by measuring the temperature drop rate after the light source is removed.

$$\tau_{s}=\frac{m_{D}C_{D}}{hA}.$$ (2)

The value of hA is obtained by measuring the temperature drop rate and calculated from Eq. 2. τ_s_ is the time constant of the system, which was calculated to be 92.8 s (Figure 4B). C_D_ (4.2 J g^−1^) and m_D_ (0.1 g) are the mass and specific heat capacity of the pure water. Q_0_ is measured independently. Therefore, the photothermal conversion efficiency of CSP core-shell nanostructures driven by 808 nm excitation can be calculated to be 52.8%, which is high enough for photothermal agents.

CSP core-shell nanostructures exhibited appropriate pore size, high pore volume, and large surface area; thus, they were promising to be used as drug carriers. The loading of uPA can be achieved by mixing and stirring the uPA and CSP core-shell nanostructures overnight. To determine the content of the loaded uPA, the drug was labeled by 5-carboxyfluorescein (5-FAM). By determining the fluorescence intensity of 5-FAM-uPA, the drug loading content was calculated to be 8.2% and the loading efficiency can be determined to be 89.6%.

**FIGURE 5:**
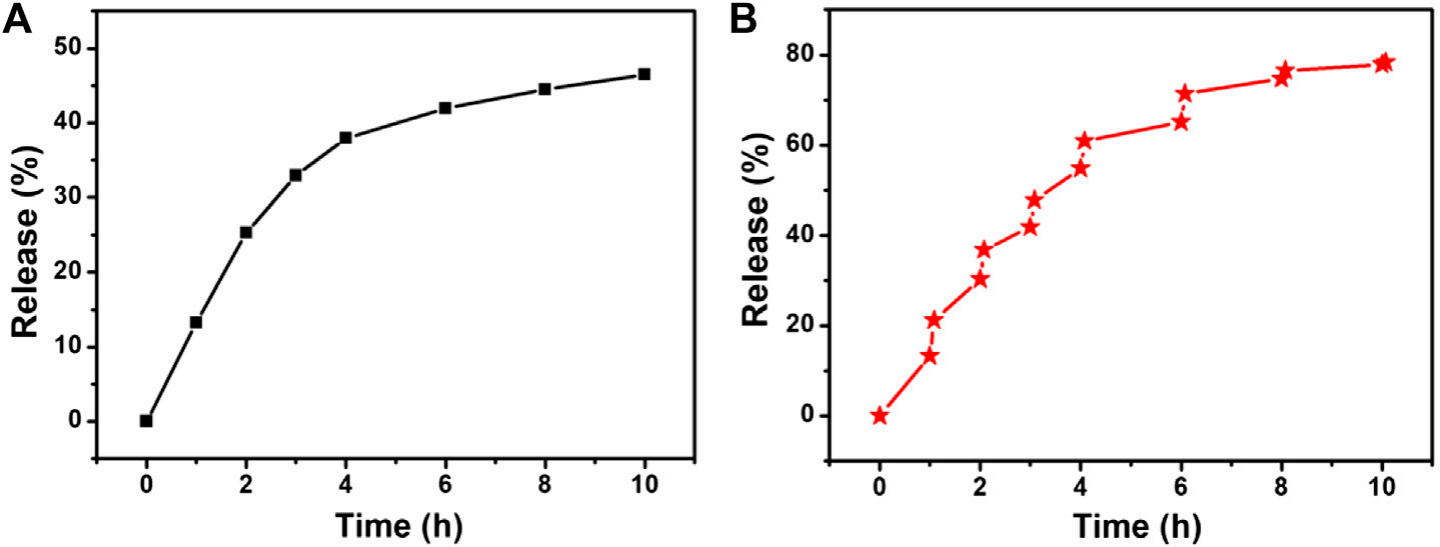
Drug release profile from the CSPA **(A)** without and **(B)** with the irradiation of the 808 nm laser.

To determine the effect of temperature changes on drug release, we studied the release of 5-FAM-uPA from CSP core-shell nanostructures against saline solution at pH 7.4 with or without the irradiation of the 808 nm laser. The determined release amount of uPA was calculated to be 11.8% for 1 h and 42.1% for 10 h without the irradiation of the 808 nm laser (Figure 5A). However, the release rate of uPA was much faster with the irradiation of the same 808 nm laser. The cumulative release of uPA was determined to be 21.4% with 1 h and 74.5% for 10 h with the irradiation of the same 808 nm laser (Figure 5B). It can be concluded that the NIR laser irradiation could accelerate the release of uPA, which resulted from the fact that the photothermal effect from the core of CSP nanostructures upon the irradiation of the 808 nm laser could disrupt the interaction between the uPA and silica shell. Therefore, the release of uPA from CSPA nanocarriers can be controlled by the NIR light.

**FIGURE 6:**
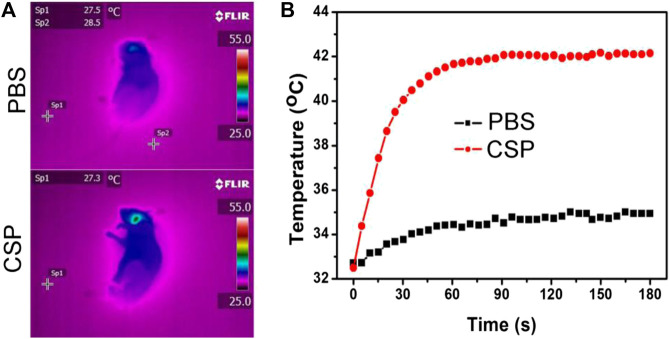
**(A)** Thermal images of the mice injected with PBS **(up)** and CSP **(down)** and then irradiated by the 808 nm lasers. **(B)** The photothermal effect *in vivo*.

The excellent photothermal effect *in vitro* of CSP core-shell nanostructures motivated us to investigate the thermal imaging *in vivo*. Before realizing the thermal imaging, we built a carotid artery thrombosis model. CSP core-shell nanostructures and PBS were intravenously injected into the mice. Twenty-four hours after the injection, the mice injected with CSP core-shell nanostructures or PBS were irradiated with the 808 nm laser (0.5 W cm^−2^, 180 s). During the laser irradiation, an infrared camera was used to record the full-body infrared thermal images. As expected, mice injected with CSP core-shell nanostructures showed thermal images with a clear contrast; as control, mice injected with PBS showed thermal images with no changes (Figure 6A). As shown in Figure 6B, the temperature of mice injected with PBS increased by less than 2 °C, while the temperature of mice injected with CSP core-shell nanostructures increased to 42 °C (Figure 6B). These results indicated that CSP core-shell nanostructures still showed excellent photothermal effect *in vivo*.

**FIGURE 7:**
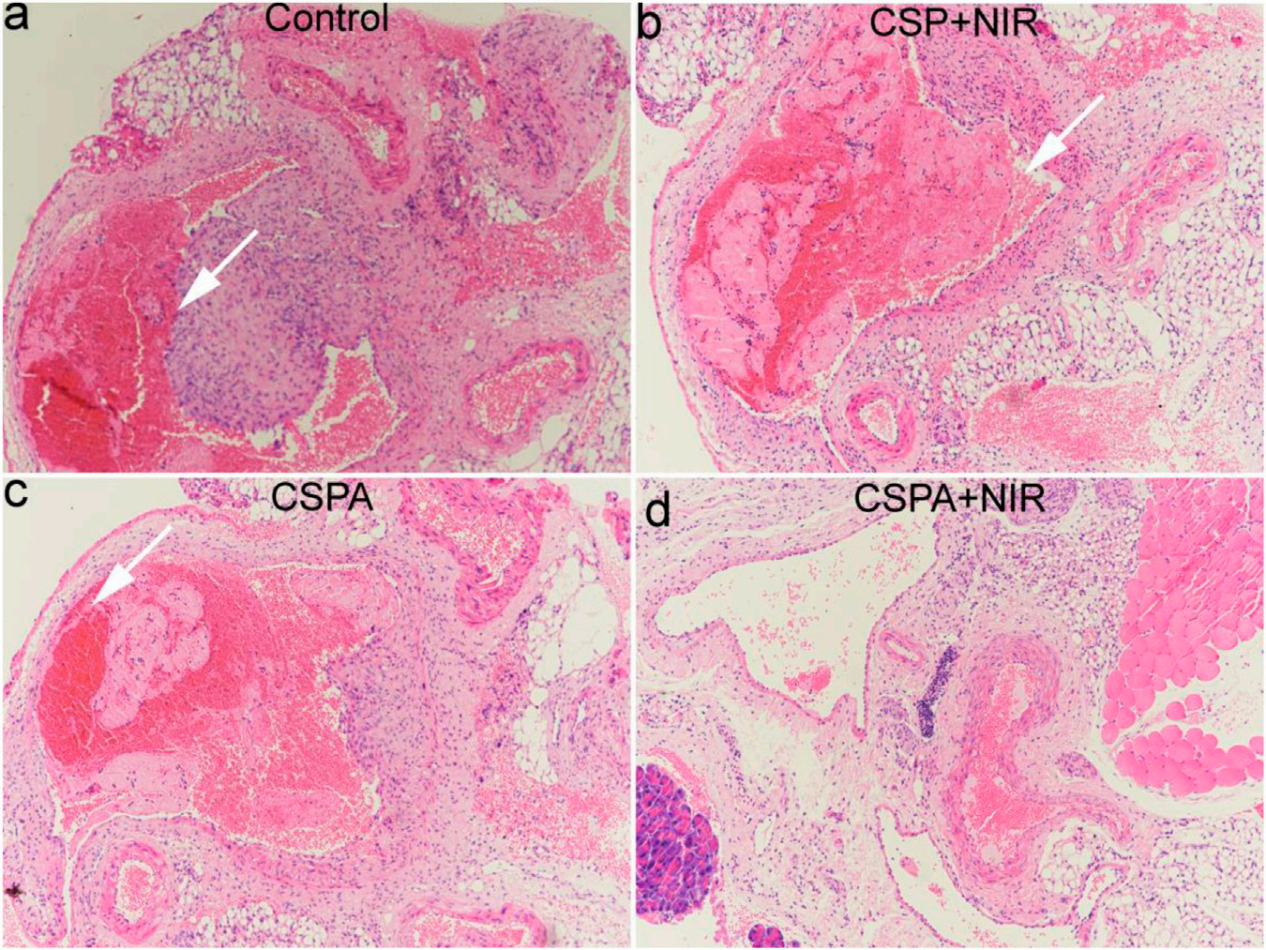
H&E staining analysis of carotid artery thrombosis after indicated treatments. **(A)** Control; **(B)** CSP + NIR; **(C)** CSPA; **(D)** CSP + NIR.

We then studied thrombolytic ability *in vivo* of CSPA core-shell nanostructures to further verify our hypothesis. The mice with the carotid artery thrombosis model were randomly divided into four groups, six mice in each group. The mice were subjected to different treatments as follows: 1) mice only irradiated with the 808 nm laser (0.5 W cm^−2^ for 5 min, Control); 2) mice injected with CSP core-shell nanostructures dispersed in saline and then irradiated with the same 808 nm laser (CSP + NIR); 3) mice injected with CSPA core-shell nanostructures dispersed in saline without NIR irradiation (CSPA); 4) mice injected with CSPA nanostructures dispersed in saline with the same 808 nm laser (CSPA + NIR). Twenty-four hours after the above-indicated treatments, carotid artery thrombosis was taken out for histological analysis. In the control group, the thrombus was filled with the carotid artery (white arrow, Figure 7A), indicating that NIR alone could not exhibit thrombolytic ability. For PTT (Figure 7B) or uPA treatment (Figure 7C), part of the thrombus was dissolved. However, for the carotid artery with thrombus treated with the injection of CSP core-shell nanostructures dispersed in saline and then irradiation with the 808 nm laser, almost no thrombus was observed. Therefore, the PTT combined with uPA showed excellent thrombolytic ability.

**FIGURE 8:**
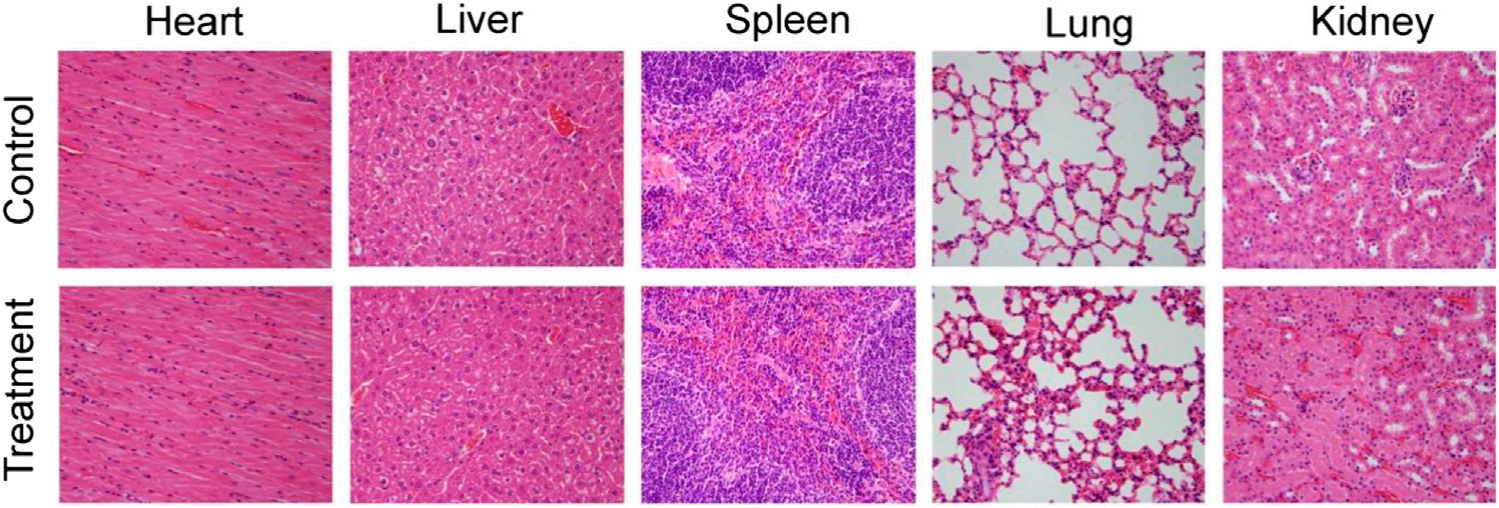
H&E-stained slices of main organs. Magnification: 400 times.

Previous work has demonstrated that inorganic nanoparticles with a size of above 10 nm would accumulate in reticuloendothelial systems (RES), such as liver and spleen (Choi et al., 2007). Thus, the CSP nanoparticles could be excreted from the body via the liver and spleen. Considering the toxicity of Cu^2+^ released from the CSP nanoparticles, the release behavior of Cu^2+^ from CSP nanoparticles in PBS was evaluated. It was found that Cu^2+^ was gradually released from CSP nanoparticles over time, but the concentration was very low (Supplementary Figure S5), showing almost no toxicity. Therefore, the CSP nanoparticles could be gradually degraded by the Cu^2+^ release from CSP nanoparticles. The long-term toxicity *in vivo* of CSP nanoparticles was evaluated by H&E analysis of major organs (heart, liver, spleen, lung, and kidney). For H&E analysis of major organs, healthy mice were injected intravenously with CSP nanoparticles with a concentration of 5 mg∙kg^−1^ (treatment group) or PBS solution (control group), mice were sacrificed and major organs were collected for H&E analysis. From Figure 8, it was observed that the shape and size of the cells from the major organs in the two groups showed almost no difference, indicating the low toxicity *in vivo* of CSP nanoparticles at the given dose.

## Conclusion

CuS nanostructures have attracted increasing attention in recent years due to low cost, high photothermal conversion efficiency, good photostability, and synthetic simplicity. The coating mSiO_2_ shell on the surface of CuS can protect the CSP nanocomposites from the external environment and thereby improve the biocompatibility and stability; also, this shell makes the CSP as NIR-triggered nanocarriers.

CSP core-shell nanoparticles with the CuS nanoparticles with the core as photothermal agents and the mesoporous SiO_2_ and the shell for the loading of uPA were successfully prepared by a simple method as the NIR light-triggered drug delivery system for thrombolysis. The CSP core-shell nanoparticles showed excellent photothermal performance, exhibiting a photothermal conversion efficiency of up to 52.8%. Due to the mesoporous SiO_2_ coating, the CSP core-shell nanoparticles exhibited appropriate pore size, high pore volume, and large surface area and thus can be used as the carrier of uPA. More importantly, the release of uPA from CSPA can be promoted by the NIR laser irradiation. Due to the excellent photothermal effect, CSP core-shell nanoparticles can be used for infrared thermal imaging *in vivo*. The *in vivo* thrombolysis assessment demonstrated that CSPA core-shell nanoparticles showed excellent thrombolytic ability under the irradiation of an 808 nm laser.

## Data Availability

The original contributions presented in the study are included in the article/Supplementary Material; further inquiries can be directed to the corresponding authors.
