# Supplementary material for: Thrombolysis Combined Therapy Using CuS@SiO2-PEG/uPA Nanoparticles
Source: Front Chem. 2021 Mar 11;9:643411. doi: 10.3389/fchem.2021.643411 (PMC7991581; doi:10.3389/fchem.2021.643411)
Supplement: Supplementary file 1 [file table1.docx]

**Thrombolysis combined therapy using CuS@SiO_2_-PEG/uPA nanoparticles**

Dapeng Fu^1^, Qingbo Fang^4^, Fukang Yuan^2, 3, 5*^, Junle Liu^1^, Heyi Ding^1^, Xuan Chen^1^, Chaoyi Cui^2,3, 6*^, Jinhui Ding^1^*

^1^ Department of vascular surgery, Karamay Central Hospital, Karamay, China.

^2^ Department of Vascular Surgery, Fengcheng Hospital of Fengxian District, Shanghai, China.

^3^Department of Vascular Surgery, Fengcheng Branch, Shanghai Ninth People’s Hospital Affiliated to Shanghai JiaoTong University School of Medicine, Shanghai, China.

^4^Department of vascular surgery, The People‘s Hospitial of Xinjiang Uygur Autonomous Redion, Urumqi,China

^5^ Department of General Surgery of XuZhou Central Hospital, XuZhou, Jiangsu, China.

^6^ Department of Vascular Surgery, Shanghai Ninth People’s Hospital, Shanghai Jiao

Tong University School of Medicine, Shanghai, China.

* Correspondence: Fukang Yuan, E-mail: jsbuck520@163.com; Chaoyi Cui, Email: cuichoi8432@163.com; Jinhui Ding, Email: 2817602110@qq.com


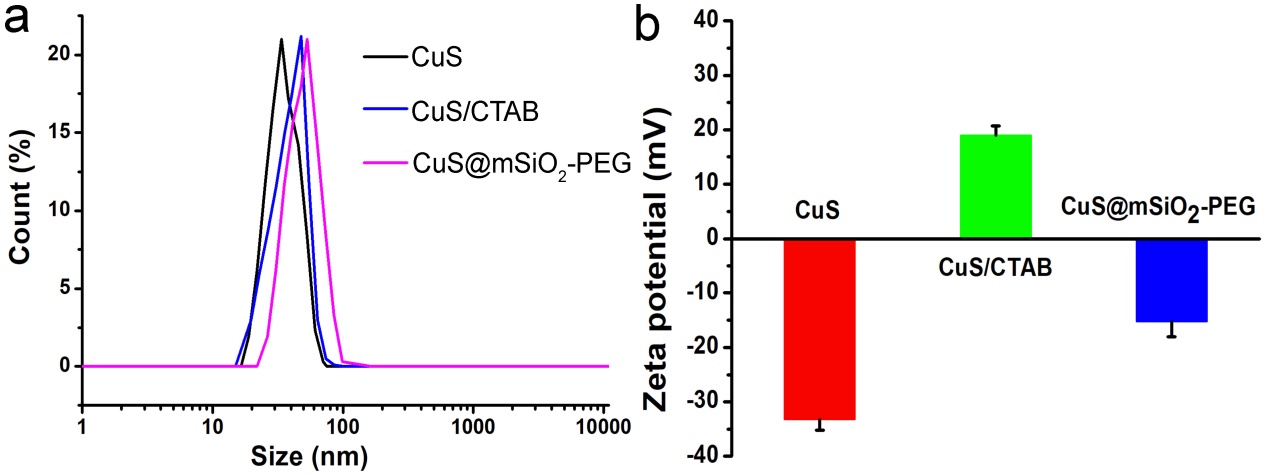


**Figure S1**. **The size (a) and zeta potential (b) of CuS, CuS/CTAB and CuS@mSiO_2_-PEG nanoparticles.**


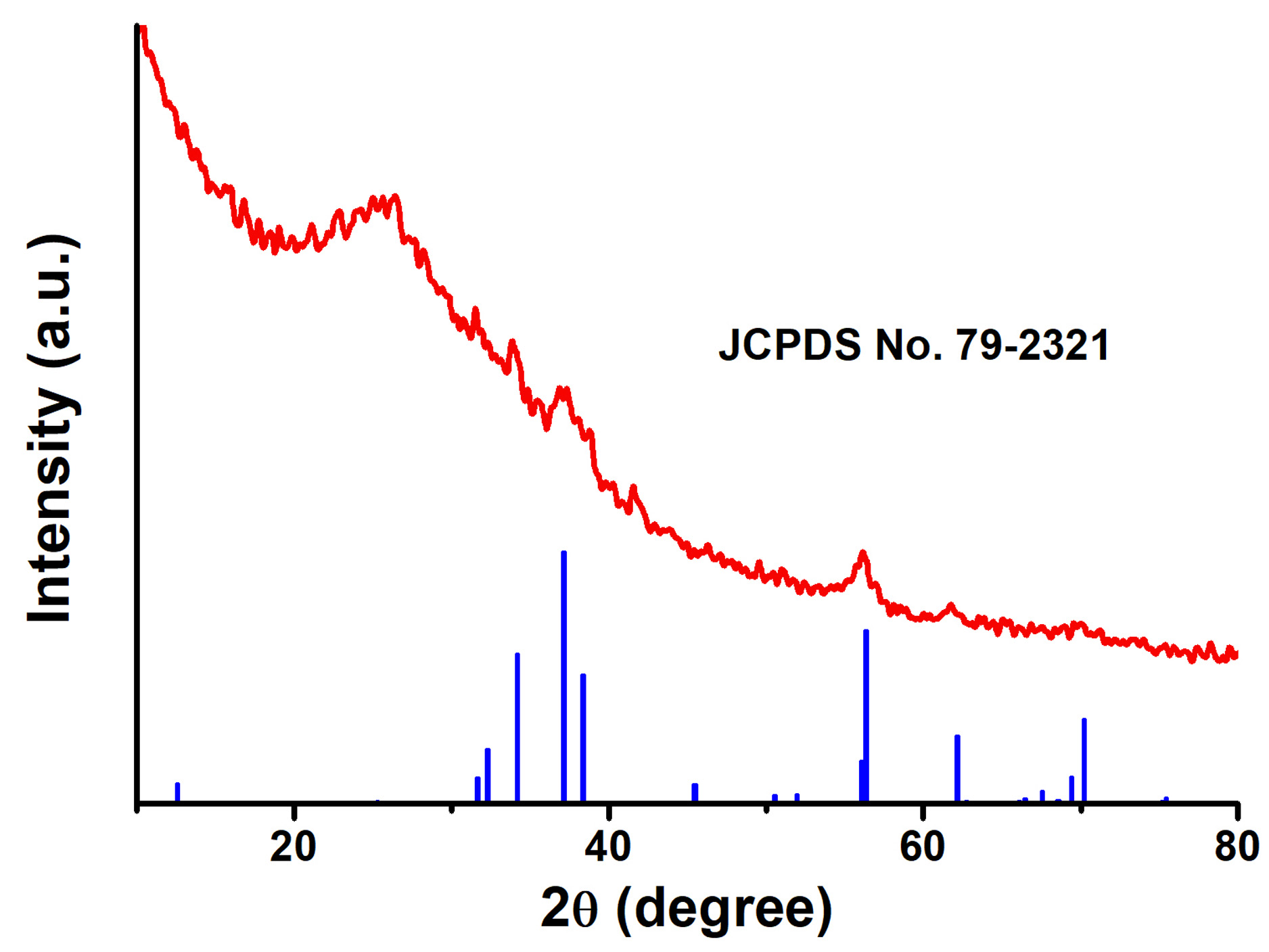


**Figure S2**. XRD pattern of CSP nanoparticles.


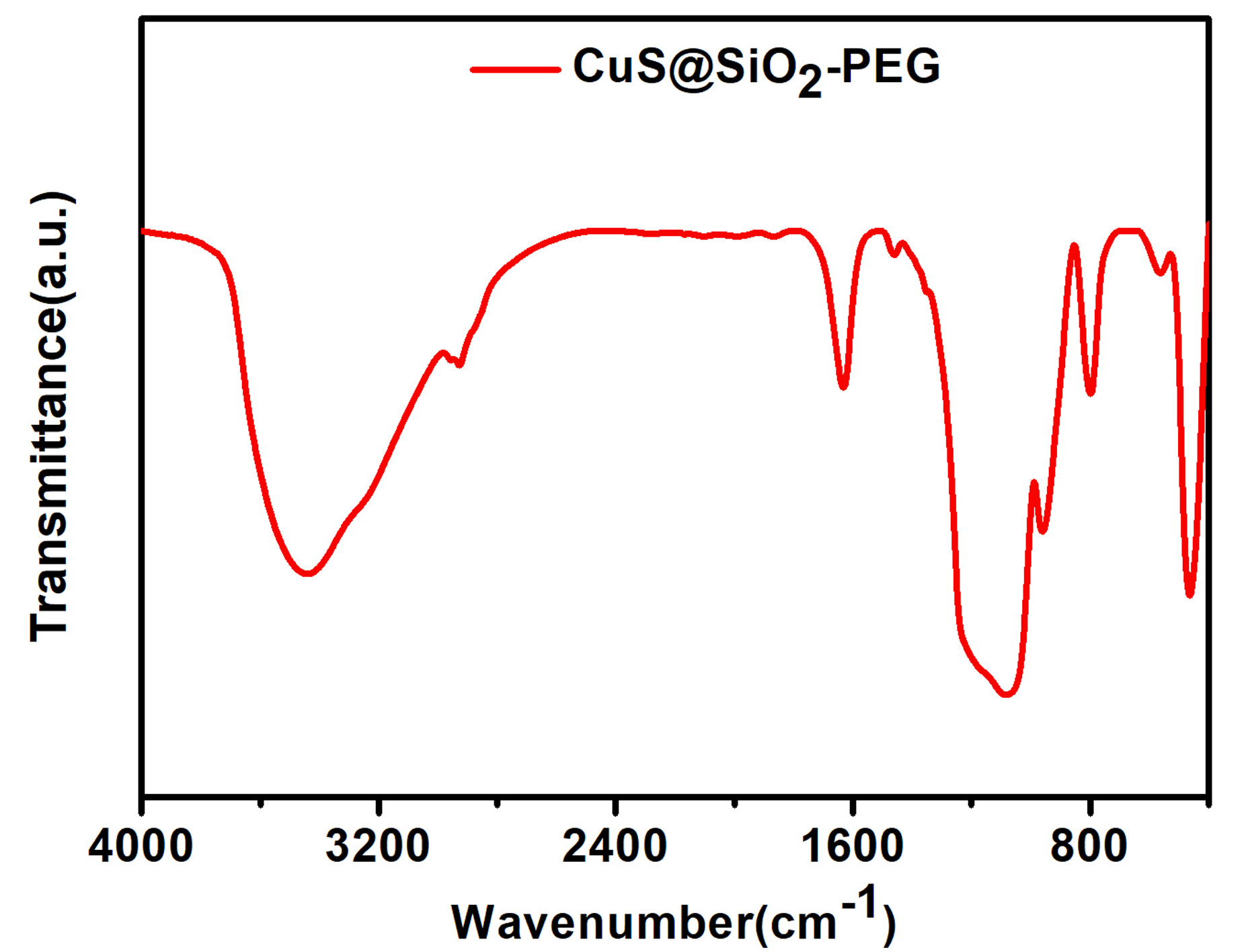


**Figure S3**. FTIR spectra of CSP nanoparticles.


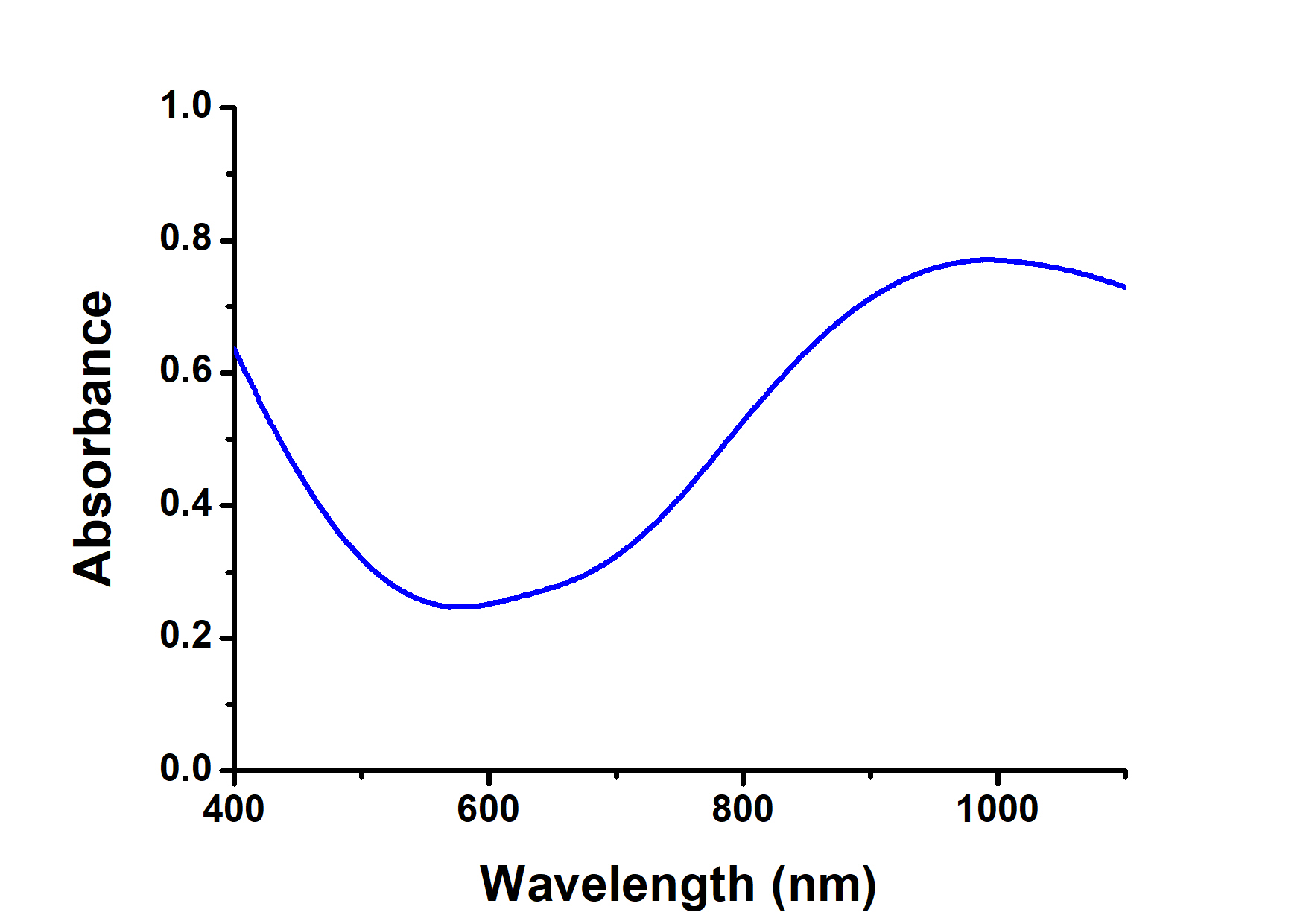


**Figure S4**. UV-vis spectra of CSP nanoparticles.


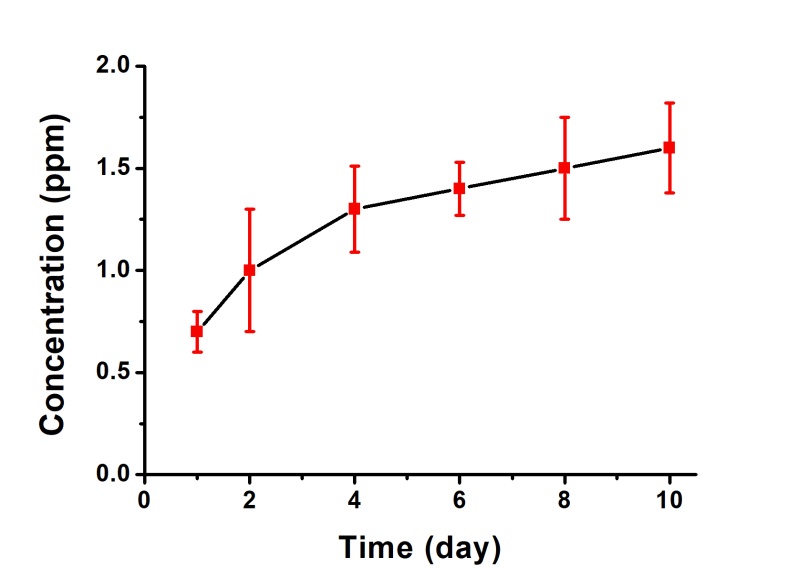


**Figure S5. The Cu^2+^ release of CuS@SiO_2_-PEG nanoparticles (3 mg/mL) in PBS.**
